# Social Network Analysis and Nutritional Behavior: An Integrated Modeling Approach

**DOI:** 10.3389/fpsyg.2016.00018

**Published:** 2016-01-29

**Authors:** Alistair M. Senior, Mathieu Lihoreau, Camille Buhl, David Raubenheimer, Stephen J. Simpson

**Affiliations:** ^1^Charles Perkins Centre, The University of Sydney Sydney, NSW, Australia; ^2^School of Mathematics and Statistics, The University of Sydney Sydney, NSW, Australia; ^3^Centre National de la Recherche Scientifique, Centre de Recherches sur la Cognition Animale Toulouse, France; ^4^Centre de Recherches sur la Cognition Animale, Université Paul Sabatier Toulouse, France; ^5^School of Agriculture, Food and Wine, The University of Adelaide Adelaide, SA, Australia; ^6^Faculty of Veterinary Science, The University of Sydney Sydney, NSW, Australia; ^7^School of Life and Environmental Sciences, The University of Sydney Sydney, NSW, Australia

**Keywords:** animal behavior, dominance hierarchy, geometric framework, nutrition, nutritional geometry, social networks

## Abstract

Animals have evolved complex foraging strategies to obtain a nutritionally balanced diet and associated fitness benefits. Recent research combining state-space models of nutritional geometry with agent-based models (ABMs), show how nutrient targeted foraging behavior can also influence animal social interactions, ultimately affecting collective dynamics and group structures. Here we demonstrate how social network analyses can be integrated into such a modeling framework and provide a practical analytical tool to compare experimental results with theory. We illustrate our approach by examining the case of nutritionally mediated dominance hierarchies. First we show how nutritionally explicit ABMs that simulate the emergence of dominance hierarchies can be used to generate social networks. Importantly the structural properties of our simulated networks bear similarities to dominance networks of real animals (where conflicts are not always directly related to nutrition). Finally, we demonstrate how metrics from social network analyses can be used to predict the fitness of agents in these simulated competitive environments. Our results highlight the potential importance of nutritional mechanisms in shaping dominance interactions in a wide range of social and ecological contexts. Nutrition likely influences social interactions in many species, and yet a theoretical framework for exploring these effects is currently lacking. Combining social network analyses with computational models from nutritional ecology may bridge this divide, representing a pragmatic approach for generating theoretical predictions for nutritional experiments.

## Introduction

Animals, from insects to human, have evolved sophisticated foraging strategies, which allow them to acquire nutrients in amounts and balances that maximize fitness (Simpson and Raubenheimer, 2012; Senior et al., 2015b). Over recent years, research in nutritional ecology has begun to reveal how these individual strategies can influence the ways animals interact within groups and populations, with far reaching consequences for collective dynamics and social structures (Simpson et al., 2006; Eggert et al., 2008; Salomon et al., 2008; Dussutour and Simpson, 2009; Bazazi et al., 2011; Lihoreau et al., 2015; Machovsky-Capuska et al., 2015). A striking example is the mass migrations of swarming crickets and locusts, where food depletion increases cannibalistic interactions and triggers the onset of the coordinated migration of tens of thousands of insects marching together in search of protein and mineral salts (Simpson et al., 2006; Bazazi et al., 2011).

**Figure 1:**
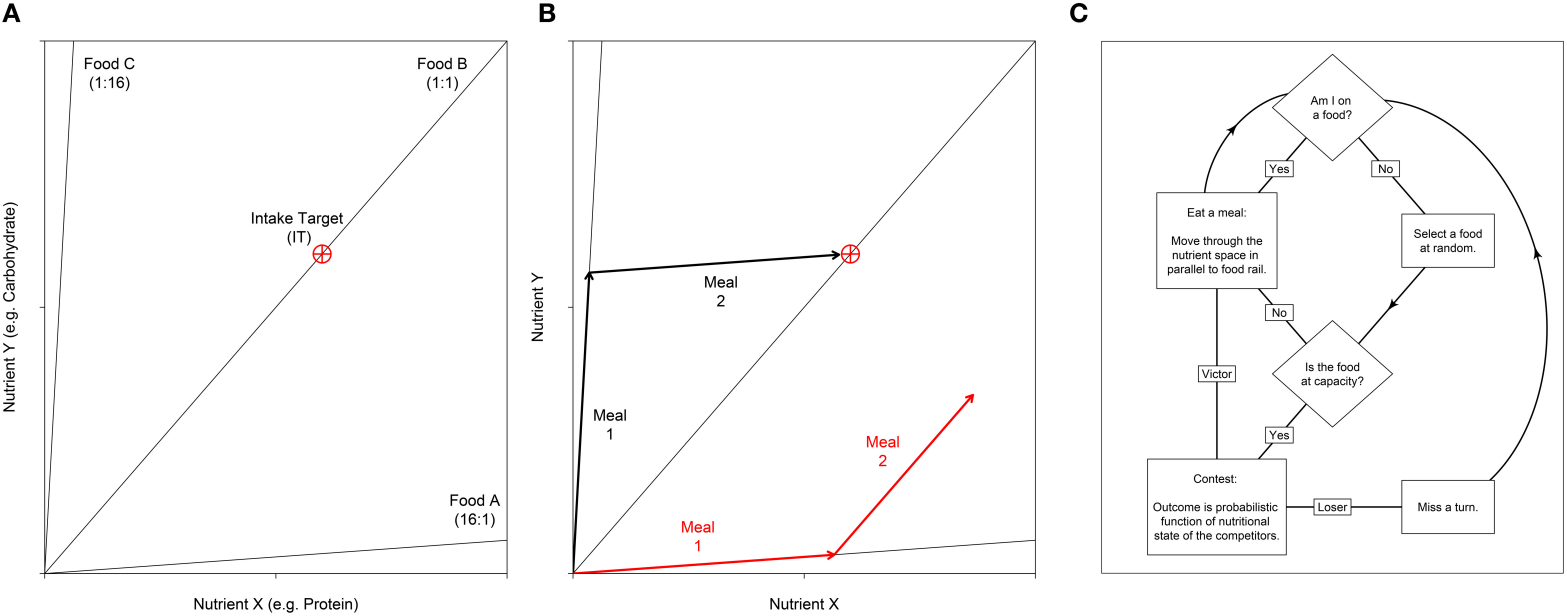
**(A)** Nutritional geometry used to model the nutrient space available for two nutrients; e.g., the macronutrients protein and carbohydrate may be represented on the *x*- and *y*- axis respectively. Foods are represented by food rails, which project through the nutrient space with a slope denoting the nutrient content for the two foods modeled. In this example the environment contains three foods. Food A is high in nutrient X relative to nutrient Y (ratio = 16:1), food B is balanced for the two nutrients and food C is low in nutrient X relative to Y (ratio = 1:16). The optimal nutritional state for an individual is represented by the Intake Target (IT), which is shown as a red crosshair. **(B)** An individual's position in the nutrient space is its (*x, y*) coordinate, which moves as the individual eats. Here we show how two different individuals move as they eat two meals. Individual 1 (black) first eats food C then food A. With respect to the IT, these two foods are nutritionally complementary and as such the individual can get close to the IT. In contrast, individual 2 (red) has first eaten food A, then food B. These two foods are not complementary, and as such the individual cannot reach the IT. Given that the IT is the optimal nutritional state, individual 2 would have a lower fitness that individual 1. **(C)** An overview of the sequence of events in the agent-based model. Individuals are processed in a random sequence. Complete code is available in Supplementary File S2. A full model description is given by Senior et al. (2015a).

Nutrient regulation strategies and their social consequences can be studied within a single modeling framework, which integrates state-space models used in nutritional geometry studies and agent-based models (ABMs) used in collective animal behavior studies (Lihoreau et al., 2014, 2015). In nutritional geometry, individuals, foods and their interactions are represented in a single nutritional space delimited by two or more nutrients (see examples in Figures 1A,B; Simpson and Raubenheimer, 1993, 2012; Simpson et al., 2015). The challenge for an individual is to eat the available foods so as to regulate its acquisition of nutrients and reach a nutritional state (NS) that maximizes fitness, known as the intake target (see examples in Figures 1A,B). Within a group or a population, these individual decisions may be complicated by social and competitive interactions, potentially creating conflicts over nutrient acquisition among the different foragers. Implementations of such nutritional geometry focused ABMs, in which multiple individuals simultaneously attempt to regulate their nutrient balance, illustrate how these simple nutritional behaviors can critically affect a range of social interactions and social structures across group types, species and ecological contexts (Lihoreau et al., 2014, 2015; Senior et al., 2015a).

Perhaps one of the best-documented examples of nutritionally mediated social structures are dominance hierarchies, where experimental studies point toward a direct role of diet in determining individual dominance and subordinate statuses (Baker et al., 1981; Whitten, 1983; McCarthy et al., 1992; Eggert et al., 2008; Salomon et al., 2008; Stears et al., 2014; Wright and Robbins, 2014). In social spiders (*Stegodyphus* sp.), for instance, the largest females of the colony tend to monopolize prey high in lipids through contest competition and thus become breeders, whereas smaller individuals that are deprived of lipids act as helpers (Rypstra, 1993; Whitehouse and Lubin, 1999; Salomon et al., 2008). In these cooperatively breeding groups, differential nutrition triggers significant skews in body size, with reproduction limited to just one or two larger females (Rypstra, 1993; Ulbrich and Henschel, 1999; Whitehouse and Lubin, 2005). Nutritional geometry focused ABMs, such as those outlined above, succinctly capture how contest competition over limited nutrients can lead to clear and stable dominance hierarchies where fitness is highly skewed toward a few individuals (Lihoreau et al., 2014; Senior et al., 2015a). In these models, access to foods is limited and individuals must engage in contests to gain a meal. The group is initially homogeneous, and early contests for access to foods have stochastic outcomes. However, after a few “contested meals,” small differences in the NS of individuals can emerge purely by chance (Senior et al., 2015a). Given the assumption that better nourished individuals outperform more poorly nourished competitors in future contests, positive feedback amplifies initial small differences in NS into a hierarchy where a few individuals monopolize nutrients, thereby gaining considerable fitness benefits. Similar mechanisms have been observed in animal dominance hierarchies arising from various types of non-nutritionally related conflicts, and are called “winner effects” (Dugatkin, 1997; Franz et al., 2015; Kura et al., 2015).

So far these models have explored the effects of the nutritional environment on individual fitness (as measured by their NS) and its variance within groups or populations. Less studied however, but of fundamental importance, is the behavior of individuals, the dynamics of their interactions and the associated consequences for social structures. Social network analyses, which are increasingly used in behavioral studies to characterize pairwise interactions between all members of a group or population (Krause et al., 2007; Croft et al., 2008; Sih et al., 2009; Sueur et al., 2011; Pinter-Wollman et al., 2013), constitute a well-developed analytical framework with which to explore the role of nutrition in mediating social interactions and their evolution. This approach is particularly powerful when it comes to characterizing complex dominance relationships in animal groups (e.g., identifying dominant individuals and how they interact with other group members based on traits such as kinship, age, sex or previous experience), and understanding the processes that underlie the network structures and dynamics (Croft et al., 2008; Shizuka and McDonald, 2012; Nandi et al., 2014).

Here we argue that social network analyses can bring fundamental new insights into research on nutritional behavior, and the consequences of these behaviors for group dynamics. We demonstrate this concept using the well-known example of nutritionally mediated dominance hierarchies. We first show that the networks generated by simple nutritional models simulating the emergence of dominance bear striking structural similarities to those networks of dominance hierarchies observed in animal groups across contexts (i.e., conflicts not necessarily related to food access). We then go on to show that metrics from social network analyses predict the future fitness of agents in these simulated competitive environments. Our analyses demonstrate how network analysis of data from these nutritional models can be used to form new empirically testable predictions for studies on social groups, bridging the gap between theoretical and experimental data.

## Methods

### Agent based model

An overview of our ABM's process is given in Figure 1C. The basal model has been previously defined in Lihoreau et al. (2014) and a detailed description (as recommended for ABMs; Grimm et al., 2006, 2010) is available in Senior et al. (2015a). The complete code, written in NetLogo version 5.1.0 (Wilensky, 1999), is available in the Supplementary File S2.

The ABM incorporates principles of nutritional geometry as described in Figures 1A,B. For simplicity we consider an environment containing just two nutrients (nutrients X and Y) in a Cartesian (*x, y*) coordinate system termed the “nutrient space.” Foods are represented as radials at angles determined by the balance of the nutrients they contain. An individual's (*x, y*) position in the nutrient space is their NS, which moves as they eat the foods that are available. At any one time only a single food can be eaten, thus individuals move their NS in parallel to the food rail that constitutes the food consumed. A single (*x, y*) coordinate, the IT, represents the point in the nutrient space that is an individual's optimal state. As such, when an individual's NS reaches the IT, fitness is maximized. Here we assume that fitness declines as the Euclidean distance between the NS and the IT increases (see Equation 6 in Senior et al., 2015a).

All individuals are initiated with the same NS (0, 0), and are given a fixed number of iterations, or “meals,” to reach the IT. Each food has a different nutritional composition, and these foods may be eaten in a number of combinations in order to reach the IT (Figures 1A,B). On each iteration, individuals attempt to eat one of the foods in the environment. However, each food has an upper limit and can thus only support a limited number of foragers. If individuals select a food that is already at this limit, they must first displace a randomly selected competitor via a dominance interaction. We began by exploring scenarios where the capacity of foods to support individuals, and thus the intensity of contest competition, was varied (manipulated via the “competition intensity” parameter *c*). Although we later focus solely on analyses of networks generated in scenarios where competition is intense (discussed further below). All data presented in the main text come from an environment containing three different foods (equivalent to that in Figure 1A). In a previous analysis of this model such a three-food environment, composed of one balanced food (i.e., individuals can reach their IT by eating only this food) and two imbalanced but complementary foods (i.e., individuals can reach their IT by mixing their intake of the two foods), produced results indicative of a wide range of environments (i.e., containing combinations of foods with different levels of nutritional complementarity; Senior et al., 2015a). Additional explorations of the model with only two imbalanced foods (equivalent to that in Figure 1A but without food B) yielded qualitatively identical results. These analyses are presented in the Supplementary Materials (Figures S1–S3).

In the model, the probability of an individual defeating another in a contest is a function of the NS of the two, as given by Equation (1):

(1) $$Q_{ij}=\frac{1}{1+e^{-\eta (F_{i}-F_{j})}}=1-Q_{ji},$$

where *Q*_*ij*_ is the probability of the *i*th individual defeating the *j*th, *e* is the natural exponent, *F*_*i*_ and *F*_*j*_ is the fitness of the two individuals, and η is a constant that scales how the difference in fitness between the two individuals governs the outcome of the contest (here fixed at 25; see Senior et al., 2015a). Accordingly, an individual with a NS closer to the IT is more likely to defeat a competitor with a NS further away. Contests between individuals with NSs close to one another have highly stochastic outcomes (this model of contest competition is based on that published by Bonabeau et al., 1996).

In previous studies involving nutritional geometry focused ABMs, the outcomes of individual interactions, have been largely overlooked. Rather, the analyses focused on the fitness and NSs of individuals after thousands of interactions, and in turn the effects that these interactions have on the evolution of nutritional strategies (e.g., Senior et al., 2015a). We here modified the model to record the outcomes of each specific individual contest at a number of different time steps with the aim of characterizing the dynamics of network growth and structure.

### Network analyses

For each simulation of the model, we constructed a time series of (after 1, 3, 5, 7, 10, 15, and 20 meals) “contest matrices.” In these matrices each row and column represents an individual, and the cells in a specific row give the total number of contests that a specific individual (given by that row) won against each other group member (in each column). Contest matrices were used to build a time series of weighted and directional networks (one for each time point), where each node represents an individual and edges represent contests between two individuals. The size of the node represents the current fitness of the individual relative to the rest of the group. The directionality of the edge denotes the outcome of the contest (arrow points toward the loser), and the weight gives the total count of contests between those individuals at that time point (i.e., previous contests are not “forgotten”). We began by using simple network visualization techniques for small, easily visualizable, social groups of seven individuals. Network graphs were implemented with the “plot.igraph” function in the *igraph* package (Csardi and Nepusz, 2006) for *R* version 3.2.1 (R Development Core Team, 2015), to demonstrate how the network and individual fitness (a function of NS), co-vary over time in different nutritional environments.

In studies of animal dominance, deconstructive approaches where networks are separated into their constitutive triads, or “motifs,” are increasingly popular tools for understanding social network structure (Shizuka and McDonald, 2012; Pinter-Wollman et al., 2013). We used this approach to compare the structures of dominance networks generated via nutritional mechanisms in our model with the typical structure of published animal dominance networks (based on the comparative results of McDonald and Shizuka, 2013). We ran replicates of the model assuming an environment where foods had a very low capacity to support individuals (i.e., where competition was intense and contests over foods frequent), and also using larger more complex social groups of 20 individuals (a group size more comparable to that of most published animal networks; McDonald and Shizuka, 2013). In these later analyses we focus solely on a food-limited environment, where competition intensity is high (*c* = 0.8). We restrict our analyses to these environments because, where foods are more abundant and competition intensity is weaker, between-agent variance in fitness is low, and strong dominance hierarchies/meaningful social structures do not arise (see Lihoreau et al., 2014).

The “contest matrices” produced by these models were converted to binary “outcome matrices,” where for any given pair of individuals the dominant is that which won the majority of contests. We also allowed for draws, or “mutual” relationships (i.e., instances where individuals won equal number of contests against one another, which were also coded as 1). Triads can take one of 16 different configurations, ranging from null triads (no interactions) to completely reciprocal relations between all three nodes, and we refer to these triads according to the Mutual Asymmetric Null (MAN) system (Holland and Leinhardt, 1970, 1976). We used the “triad.census” function in the *R* package *statnet* (Handcock et al., 2008, 2015) to get counts of each type of triad in each network. We also simulated 1000 equivalent random matrices (using the “rguman” function in *statnet*), to estimate the difference in occurrence of each triad type between our ABM-derived networks and random networks with the same number of nodes, edges and null dyads (similar to the method of McDonald and Shizuka, 2013, although they did not include draws). Whilst it is clear that differences between our networks and random networks are to be expected (e.g., we know that nutritionally derived networks are based on winner effects), we were primarily interested to see whether our simulated networks differ from random expectations in similar ways to published animal dominance networks. We also calculated the triangle transitivity (*t*_tri_) of networks, a statistic that denotes the “orderliness” of the group where 0 indicates a completely non-linear group (equivalent to a random network) and 1 a perfectly ordered linear hierarchy (McDonald and Shizuka, 2013). We calculated *t*_tri_ following the method described in Appendix 2 of Shizuka and McDonald (2012).

Finally, we assessed whether individual network statistics applied to dominance networks can be used to predict the final fitness of individuals in a social group (again using 20 individuals). To do this, we generated a weighted directional network at a number of time points as above (based on contest matrices and again where competition was intense). We calculated the (normalized) closeness of each individual (node) within the network at a given time with the “closeness_w” function in the *R* package *tnet* (Opsahl, 2009). In weighted directed networks such as which we generated, the closeness of a node is a function of: (1) the direction between two nodes (implying that the shortest path between nodes *i* and *j* is not necessarily equal to the shortest path between nodes *j* and *i*), (2) the number of edges a node has, and (3) the weight of those edges (Opsahl et al., 2010). The tradeoff between the influence that these last two characteristics have on closeness is governed by the tuning parameter α (Opsahl et al., 2010). Where α is zero, only the number of edges influence node closeness; where α is between 0 and 1 both the weight and the number of edges positively influence closeness; where α = 1 closeness depends solely on the sum of the weight of edge weights; and where α > 1 higher weights positively influence closeness and increasing numbers of edges reduce closeness (Opsahl et al., 2010). We explored closeness calculated via a number of different α-values (0, 0.5, 1, 1.5, and 2) and their biological interpretation is discussed further in the results. We then compared individual closeness to other predictors of final fitness, using linear regression implemented with the “lm” function in the *base* package in *R*. In these models the response was the fitness of the individual after 20 meals logit transformed for model fitting (fitness is bound at 0 and 1 in our models; Warton and Hui, 2010), and back-transformed for plotting.

## Results

**Figure 2:**
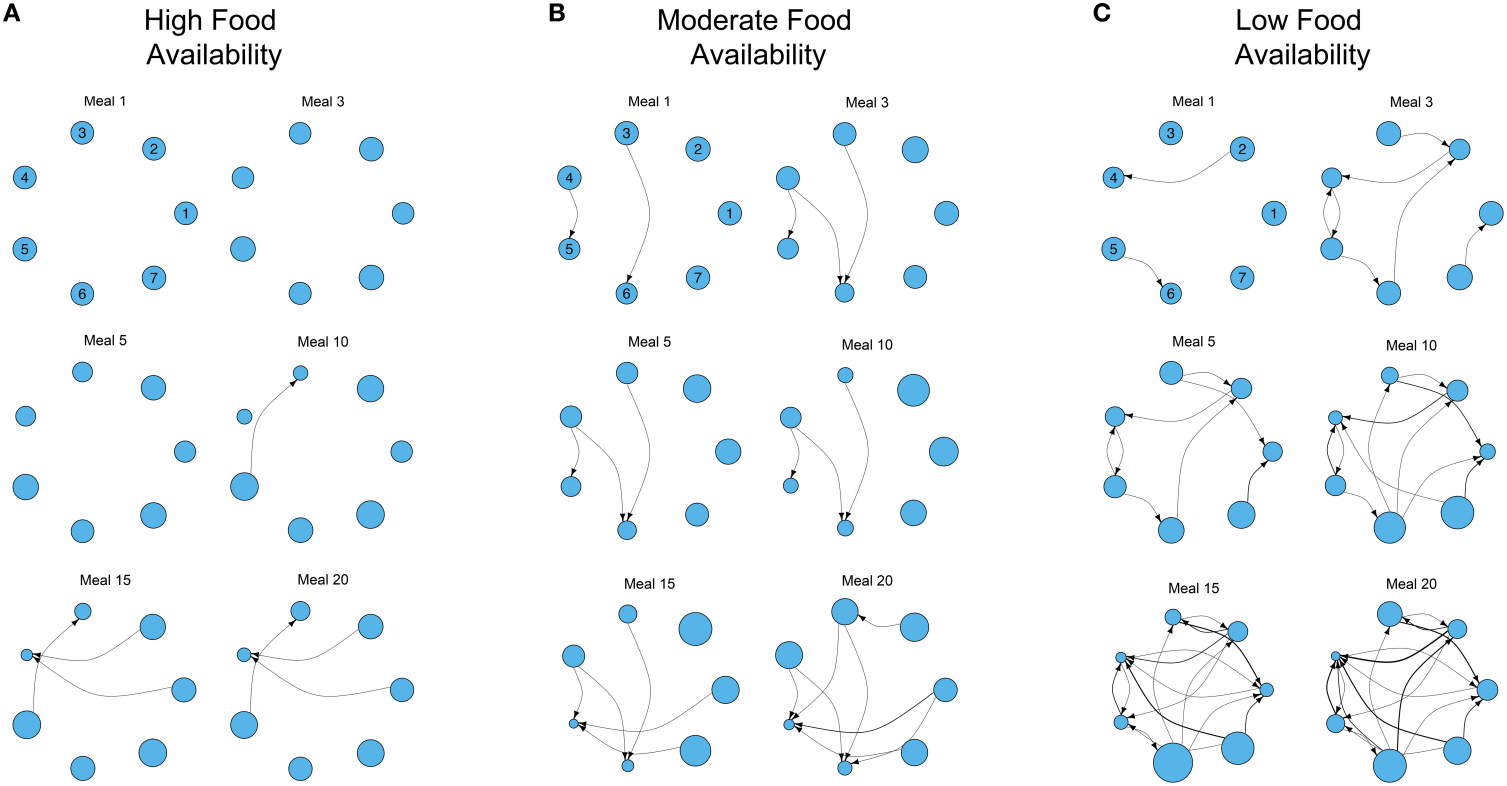
**Examples of the development of three different dominance networks in three different nutritional environments containing three foods (identical to Figure 1A) at (A) high, (B) moderate, and (C) low food availability after 1, 3, 5, 10, 15, and 20 meals, in groups of 7 individuals**. Each network is directional and weighted. Each node represents 1 of 7 individuals, and the plotted position of each individual remains constant across meals in each environment (individual identities are given for meal 1). Edges represent contests, with the direction of the edge denoting the victor of the contest and the loser (recipient). Edges are weighted by the number of contests. The size of each node represents individual fitness relative to the rest of the group (larger nodes denote fitter individuals, that have nutritional state closer to the IT). The degree of food availability equates to a “competition intensity” (*c*) of 0.4, 0.6, and 0.8, as described in Senior et al. (2015a). The script is available in Supplementary File S2.

Figure 2 shows examples of the development of three dominance networks over time in different three-food environments (i.e., identical to Figure 1A) where foods are either abundant, moderately available or scarce. The same analyses for two-food environments (i.e., foods A and C in Figure 1A) yielded qualitatively similar results (Figures S1–S3).

Where foods are more abundant, there are relatively few contests to gain access to foods. As a consequence the network is poorly connected even after 20 meals (Figure 2A). However, as food availability decreases, individuals must engage in contests to gain food access and networks become more connected (Figures 2B,C). A direct consequence of this is the increase of variance in the relative fitness of individuals within the group (variance in the size of the nodes in the network; Figures 2B,C). In the three environments all group members initially have the same fitness, but where food is less available variance in fitness gradually emerges and increases as contests and meals accumulate. For instance, in the low food environment, individual 4 has already lost multiple contests after only 10 meals (Figure 2C). After 20 simulated meals, this individual has lost a large number of contests, has the lowest fitness and a clear fitness skew has emerged. In this environment, individual 6 is the fittest, and the difference between individuals 4 and 6 is large (individual 4 is only 26.67% as fit as individual 6; Figure 2C). In contrast, where foods are relatively abundant, and networks are less connected (i.e., fewer contests), the difference between the fittest and least fit individuals in the group is less pronounced (the least fit individual is only 50.77% as fit as the fittest; Figure 2A).

**Figure 3:**
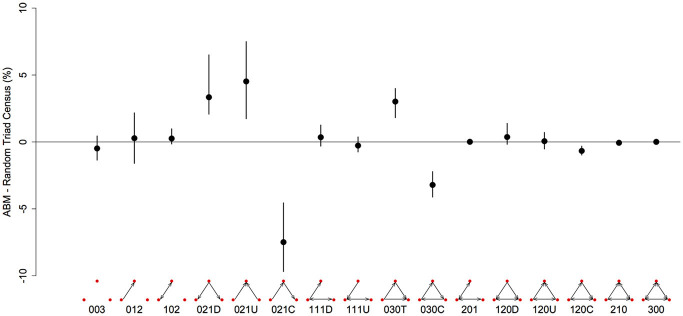
**Differences in the representation (%) of triad types between networks generated from the agent-based model and random networks with similar properties (same number of nodes, edges and null dyads) in a three-food environment (identical to Figure 1A)**. We created 10 different networks for groups of 20 individuals using our model, with a high level of “competition intensity” (*c* = 0.8) and after 20 meals. For each network we calculated the difference between itself and 1000 randomly generated networks, and took the mean of these 1000 differences. These results give the mean of the 10 sets of mean differences (black dots) and the 0.025–0.975 quantiles of these 10 (black bars). Triads are labeled according to the MAN labeling system (Holland and Leinhardt, 1970, 1976).

There are 16 possible configurations of triads in networks that are both directional and contain mutual ties (Figure 3). In our simulated dominance networks in competition-intense environments (*c* = 0.8), the most frequent types of triads were 012, 030T, 021U, 021D, and 021C (Table 1, see also Figure 3 for visualizations of these triad types). However, these results are most informative when compared to “null expectations” derived from random networks. Figure 3 displays the difference between the triad census (% of each type of triad) of ABM derived networks and random networks. There were on average 4.52 more 021U (i.e., the difference in the % of 021U triads in ABM networks and random networks was 4.52), 3.34 more 021D, and 3.01 more 030T triads than would be expected purely by chance. In addition, there were on average 7.47 less 021C (even though among the most common type of triads in our simulated networks; Table 1) and 3.20 less 030C triads in the simulated dominance networks than would be expected in a random network (Figure 3; note that 0.95 quantiles for these differences exclude 0). The mean *t*_tri_ of these networks was 0.77.

**Table 1:** **Mean (and standard deviation, SD) counts of each type of triad based on the triad censuses of 10 networks generated by our agent-based model with a high level of “competition intensity” (*c* = 0.8) and after 20 meals**.

	**Triad Type**															
	**003**	**012**	**102**	**021D**	**021U**	**021C**	**111D**	**111U**	**030T**	**030C**	**201**	**120D**	**120U**	**120C**	**210**	**300**
Mean	78.70	331.4	22.90	145.6	159.0	129.8	29.50	22.40	176.5	10.80	1.800	12.40	8.900	8.800	1.400	0.100
*SD*	15.35	14.93	11.05	18.87	23.39	17.13	10.05	6.950	27.18	5.490	1.320	6.700	5.320	4.370	1.430	0.320

*The 16 possible triad types are labeled according to the MAN system (Holland and Leinhardt, 1970, 1976), and visualizations of each type are given in Figure 3.*

To assess whether network metrics could be used to estimate an individual's performance and hierarchical rank, we examined how well closeness centrality after 5, 7, 10, and 15 meals predicted future fitness after 20 meals in environments where competition was intense, and between-individual variance in fitness high. We first explored how the tuning parameter α influenced the power of closeness to predict future fitness. At most time points (meals), linear models fitting closeness with α > 0 had higher *R*^2^ than those with α = 0 (Table 2). In addition, at early time points α-values of 0.5, 1, and 1.5 tended to have better fit than α = 2. These findings indicate that both the number of conspecifics an individual has defeated in contests (i.e., number of edges), and the number of times it does so (i.e., weight of edges) influence future fitness (as opposed to only one of these factors being of relevance). For instance a strategy whereby an individual dominates one or two conspecifics a large number of times (large α) early on in the simulation, may not be better than dominating all other conspecifics in a similar number of contests (α ~ 1).

**Table 2:** *****R***^**2**^ from linear models estimating fitness after 20 meals (logit transformed) as a function of closeness and fitness at 5, 7, 10, and 15 meals when there is a high level of “competition intensity” (***c*** = 0.8)**.

**Meals**	**Closeness**					**Current fitness**
	***α =* 0**	***α =* 0.5**	***α =* 1**	***α =* 1.5**	***α =* 2**	
5	0.281	0.297	0.311	0.319	0.314	0.631
7	0.421	0.433	0.437	0.429	0.404	0.837
10	0.528	0.537	0.536	0.516	0.473	0.884
15	0.590	0.629	0.666	0.693	0.710	0.971

*Closeness was calculated using α of 0, 0.5, 1, 1.5, and 2 (see main text), and we see how these values affect the power of closeness to predict final fitness. Higher R^2^ is indicative of better model fit.*

**Figure 4:**
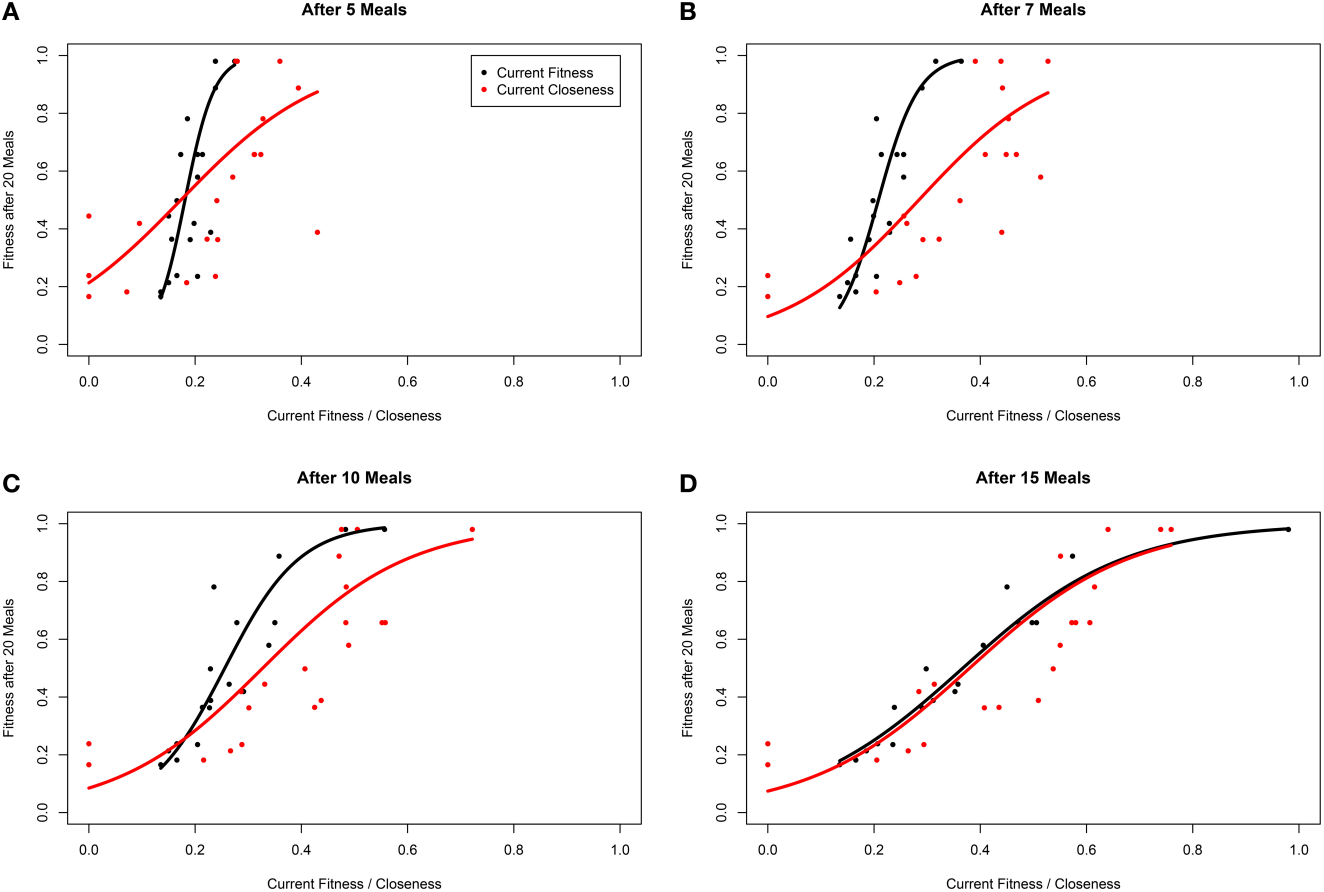
**Fitness of individuals within a simulation after 20 meals, as a function of current fitness (black) and network closeness (red), after (A) 5 meals, (B) 7 meals, (C) 10 meals, and (D) 15 meals for groups of 20 individuals in a three-food environment (identical to Figure 1A)**. The networks are based on an environment with a high level of “competition intensity” (*c* = 0.8). Curves are linear model estimates of fitness after 20 meals, as predicted by current closeness or fitness. Note that fitness after 20 meals was logit transformed for model fitting as fitness is bound at 0 and 1, before being back-transformed for plotting. Closeness values presented were calculated using α = 1 (see Opsahl et al., 2010).

Within our ABM, mechanisms of winner effects based on current NS (fitness) operate. Thus, in theory the best predictor of final position in the dominance hierarchy at any given time point should be current fitness. Accordingly, closeness (calculated with any α-value) did not appear to be as good a predictor of future fitness as current fitness (i.e., *R*^2^ for models fitting closeness were consistently lower than those fitting current fitness; Table 2). However, closeness values did correlate strongly with future fitness (Table 2 and Figure 4). Importantly, at earlier time points, between-individual variance in closeness was much greater than between-individual variance in current fitness (Figures 4A–C). Thus, closeness is a valuable predictor of future fitness in experimental studies on nutritionally mediated dominance hierarchies, as it is easy to quantify from behavioral data and less sensitive to measurement inaccuracies that NS.

## Discussion

We combined social network analyses with ABMs of nutritional geometry to generate new insights into the role that nutrition can play in the formation of dominance hierarchies in animal groups. We found that network graphs based on contest matrices constitute succinct tools for characterizing the emergence of dominance hierarchies and for comparing those hierarchies across simulated nutritional environments and through time. As a well-established and widely implemented analytic framework (Krause et al., 2007; Croft et al., 2008; Sih et al., 2009; Sueur et al., 2011; Pinter-Wollman et al., 2013), social network analysis thus represents a promising tool for investigating the role of nutrition in mediating social interactions in animal groups.

One of the main advantages of nutritional geometry focused ABMs is that their predictions can also be tested using established experimental protocols from the same framework (Simpson and Raubenheimer, 1993, 2012; Simpson et al., 2015). For instance, our models predict a relationship between current and future NS of individual animals, and consequently their future position in a dominance hierarchy as well as their fitness relative to the rest of group (Lihoreau et al., 2014). A specific difficulty of evaluating such predictions is the logistical problem of quantifying NS/fitness in social groups that are relatively “young” (recently formed groups where few social interactions have occurred). Specifically, between-individual variance in fitness will likely be low at early stages (Figures 2, 4), making accurate quantifications of the relative state of individuals difficult. In addition, without manipulative or lethal approaches, quantifying between-individual differences in NS is hard. Here, we have shown that simple measures of network centrality, such as closeness, can be used as good predictors of future fitness. These global network metrics, among others, are easier to experimentally quantify than NS or fitness, and can be calculated based on behavioral data gleaned from simple group observations. A side note is that integrating weights in to analyses of the dominance networks that we generate here is of some importance, having consequences for the biological interpretation of the formation of a dominance hierarchy. However, accurately assessing the importance to ascribe to weights in calculations of closeness (i.e., which α-values is most appropriate) requires a detailed and systematic examination of the parameter space.

Local metrics are also very informative. Motif analyses revealed significant excesses of certain triads within the dominance networks generated by our model relative to random networks. Specifically, we found that triads with transitive properties (i.e., that indicate orderliness, or a linear hierarchy) were over-represented in our model. In contrast cyclical triads (A dominates B, who dominates C, who dominates A), which indicate the lack of a clear hierarchy were under-represented. Comparative analyses, which collate data from many different forms of social conflict (i.e., the proximate cause of conflicts was variable) and across taxa, have revealed similar excesses and deficits of these same transitive triads in real world animal dominance networks (McDonald and Shizuka, 2013; Shizuka and McDonald, 2015). In addition, previous analyses of dominance networks in animals have shown an excess of “double dominant” (021D) and a lack of “pass along” (021C) triads to be common in the early stages of hierarchy formation (Chase and Rohwer, 1987; Shizuka and McDonald, 2015). We repeated our analyses after only seven (out of a possible 20) simulated meals and found similar properties among these early networks (Figure S4).

The estimated transitivity of networks generated by our model was high (*t*_tri_ = 0.77), although lower than previous comparative studies of empirically determined animal hierarchies (0.88; McDonald and Shizuka, 2013). One possible explanation is the frequency of mutual ties in our networks (i.e., A and B dominate each other an equal number of times), which are known to be rare in animal networks, and have been excluded in calculations of transitivity in previous analyses (McDonald and Shizuka, 2013; Shizuka and McDonald, 2015). We can identify two non-mutually exclusive explanations for the abundance of mutual ties in our networks. Firstly, if previously published studies are based on hierarchies that were well established at the time of observation, they may overlook very early interactions between individuals, which can have stochastic outcomes as the hierarchy has a poor linear formation. Secondly, in our model individuals pick competitors at random, and individuals do not identify another's state, or their contest history with other conspecifics (i.e., bystander effects; Dugatkin, 2001). Future inclusions of mechanisms of social recognition in our models, such as status or individual recognition (Barnard and Burk, 1979), may lead to fewer mutual ties and an increased overall triangle transitivity.

Because our approach is grounded into nutritional ecology, it is perhaps one of a handful of methods with which it is possible to generate theoretical networks based on an explicit biological mechanism (i.e., interactions governed by NS). Previously, random networks with desired structural properties have been generated using tools based on graph theory, where the underlying algorithm for generating the network is entirely dependent on the final desired properties thereof. Perhaps the most popular mechanism of non-random network generation in biology remains the preferential attachments model, where nodes are more likely to generate edges with “more popular” nodes (i.e., those with the most edges; Barabási and Albert, 1999; Akbaş et al., 2015; Carletti et al., 2015; Zuev et al., 2015). Whilst preferential attachment models seem realistic for interactions involving communication and/or cooperative behavior, they seem less applicable to animal interactions related to nutrition (e.g., predation, food webs, or contests over food access). In contrast to the standing paradigm, using our models it is possible to generate networks from the bottom up, starting with an explicit (and fully testable) nutritional mechanism, which then gives rise to individual interactions and subsequent emergent network structures (apparently similar to those properties observed in animal groups). To our knowledge, this is the first time such an approach has been documented.

Social network approaches have become increasingly popular in behavioral and ecological research, enabling extensive analyses of simultaneous interactions among multiple individuals and across long periods of time (Krause et al., 2007; Croft et al., 2008; Sih et al., 2009; Sueur et al., 2011; Pinter-Wollman et al., 2013). Our study now illustrates how this approach can benefit research on nutritional behavior, ultimately helping to elucidate complex interactions between the environment, the nutritional strategies of individual animals and the consequences thereof for social interactions and collective phenomena. Beyond the example of nutritionally mediated dominance hierarchies, the broader integration of social network analyses into nutrition research has potential for investigating the nutritional ecology of species exhibiting a great diversity of social forms, from temporary aggregations of feeding animals to permanent and fully eusocial colonies of cooperatively foraging nestmates (Krause and Ruxton, 2002). These interactions may include several types of nutrient driven social networks, including social and competitive interactions among foragers (as in this study), transfer of social information about food resources, and exchange of foods (or specific nutrients) or microorganisms (symbionts or pathogens) between individuals. Predominantly, behavioral research utilizing network analyses has focused on descriptive approaches that identify the structure of animal interactions. However, recent developments show that network approaches can be exploited to study the temporal dynamics and the function of interaction patterns (Pinter-Wollman et al., 2013). Thus, network approaches may be used to study both the causes of complex nutritional strategies (i.e., modeling social interactions that influence the nutritional behavior of individuals; e.g., Senior et al., 2015a) and their associated consequences (as in this study). Here, we have dealt with networks and social structures that arise from entirely homogenous groups, but this need not be the case and the ABM may be initialized with a heterogeneous group. For example, each individual may express one of several different nutritional strategies (e.g., young and adults, males and females, producers and scroungers, healthy or diseased animals, or individuals with differing gut microbiota; Flint et al., 2015), allowing one to explore the role that such traits play in governing the emergence of complex social structures. Importantly, our geometric approach identifies explicit nutritional mechanisms, raising the possibility of generating system-specific, empirically testable, predictions about network formation in different nutritional environments and animal groups. Ultimately, a more detailed assessment of the nutritional basis of social behavior, as afforded by social network analyses, will inform our understanding of how nutrition can drive the diversity of social forms observed in nature, a major challenge for future research in nutritional and behavioral ecology (Lihoreau et al., 2015).

## Author contributions

AS, ML, and SS conceived and designed the study. AS, CB, and ML collected and analyzed the data. All authors contributed to the writing of the manuscript. AS and ML contributed equally to this work.

### Conflict of interest statement

The authors declare that the research was conducted in the absence of any commercial or financial relationships that could be construed as a potential conflict of interest.
