# Supplementary material for: Social Network Analysis and Nutritional Behavior: An Integrated Modeling Approach
Source: Front Psychol. 2016 Jan 29;7:18. doi: 10.3389/fpsyg.2016.00018 (PMC4731493; doi:10.3389/fpsyg.2016.00018)
Supplement: Supplementary file 1 [file DataSheet1.docx]

# Supplementary File S1: Supplementary Results

## Figure S1.

Examples of the development of three different dominance networks in two-food environments containing unbalanced but complementary foods (foods A and C in Figure 1.A.). We explored three different nutritional environments (high, moderate and low food availability) after 1, 3, 5, 10, 15 and 20 meals, in groups of 7 individuals (i.e. equivalent of Figure 2, but in a two-food scenario). Each network is directional and weighted. Each node represents 1 of 7 individuals, and the plotted position of each individual remains constant across meals in each environment (individual identities are given for meal 1). Edges represent contests, with the direction of the edge denoting the victor of the contest and the loser (recipient). Edges are weighted by the number of contests. The size of each node represents individual fitness relative to the rest of the group (larger nodes denote fitter individuals, that have nutritional state closer to the IT). As food availability decreases networks become more connected (more edges) and variance in the relative fitness of individuals increases (greater differences in the size of nodes).

## Figure S2.

Differences in the representation (%) of triad types between networks generated from the agent-based model and random networks with similar properties (same number of nodes, edges and null dyads) in a two-food environment containing unbalanced but complementary foods (foods A and C in Figure 1.A.). We created 10 different networks for groups of 20 individuals using our model, with a high level of ‘competition intensity’ (*c*  = 0.8) and after 20 meals. For each network we calculated the difference between itself and 1000 randomly generated networks, and took the mean of these 1000 differences. These results give the mean of the 10 sets of mean differences (black dots) and the 0.025 to 0.975 quantiles of these 10 (black bars). Triads are labeled according to the MAN labeling system (Holland and Leinhardt, 1970; Holland and Leinhardt, 1976). Simulated networks are characterized by more 021D, 030T and 120D, and less 021C, 030C and 120C than expected in a random network. The mean t_tri_ of these simulated networks is 0.62.

## Figure S3.

Fitness of individuals within a simulation after 20 meals, as a function of current fitness (black) and network closeness (red), after A) 5 meals, B) 7 meals, C) 10 meals and D) 15 meals for groups of 20 individuals in a three-food environment (foods A and C in Figure 1.A). The networks are based on an environment with a high level of ‘competition intensity’ (*c*  = 0.8). Curves are linear model estimates of fitness after 20 meals, as predicted by current closeness or fitness, and the *R*^2^ of each model is given in the bottom right. Note that fitness after 20 meals was logit transformed for model fitting as fitness is bound at 0 and 1, before being back-transformed for plotting. Closeness was calculated using *α* = 1 (see Opsahl et al., 2010).

## Figure S4.

##

Triad motif analyses of networks (equivalent of Figure 3) after seven meals (i.e. in the earlier stages of hierarchy formation) in a three-food environment (identical to Figure 1.A). Differences in the representation (%) of triad types between networks generated from the model and random networks with similar properties (same number of nodes, edges and null dyads). We created 10 different networks for groups of 20 individuals using our model, with a high level of ‘competition intensity’ (*c*  = 0.8). For each network we calculated the difference between itself and 1000 randomly generated networks, and took the mean of these 1000 differences. These results give the mean of the 10 sets of mean differences (black dots) and the 0.025 to 0.975 quantiles of these 10 (black bars). Triads are labeled according to the MAN labeling system (Holland and Leinhardt, 1970; Holland and Leinhardt, 1976). Note the lack of 021C triads.

## Supplementary File S2: Netlogo Code

Netlogo code for the agent based model, used to generate networks.
