# Supplementary material for: Social Network Analysis and Nutritional Behavior: An Integrated Modeling Approach
Source: Front Psychol. 2016 Jan 29;7:18. doi: 10.3389/fpsyg.2016.00018 (PMC4731493; doi:10.3389/fpsyg.2016.00018)
Supplement: Supplementary file 2 [file DataSheet2.zip › File S2 Model Code.rtf]

;;; This is NetLogo code written to accompany Senior et al. 2015. Social Network Analyses and Nutritional Behaviors: An Integrated Modeling Approach;;; The code was written by A. M. Senior @ the University of Sydney in 2015;;; For the most systematic and full description of this model see ;;; Senior, A.M., Charleston, M.A., Lihoreau, M., Buhl, J., Raubenheimer, D., and Simpson, S.J. (2015). ;;; Evolving nutritional strategies in the presence of competition: a geometric agent-based model. ;;; PLoS Computational Biology 11, e1004111.;;; One key difference between this model and that described by Senior et al. (2015) is that here we include no evolutionary algorithm.;;; A second paper which is of interest is Lihoreau, M., Buhl, J., Charleston, M.A., Sword, G.A., Raubenheimer, D. and Simpson, S. J. (2014);;; Modelling nutrition across organizational levels: From individuals to superorganisms ;;; Journal of Insect Physiology 69, 2-11.;;; To run this code;;; 1) Download Netlogo (https://ccl.northwestern.edu/netlogo/);;; 2) Copy and paste all of this code under the 'code' tab;;; 3) YOU MUST also create the following on the interface tab ;;; Three sliders called 'FOOD.A', 'FOOD.B' and 'FOOD.C', with range 0 - 16.;;; These sliders manipulate the nutrient content of the food.;;; One slider called 'a' bound at 0 and 3 (or the upper bound being equivalent to the number of foods in the environment).;;; This slider allows you to control the abundance of foods - it is also useful to include a monitor called 'c' for competition, which evaluates to c = 1 - (a / the number of foods). ;;; In the MS we use a values to give c = 0.4, 0.6 and 0.8 as high, moderate and low food availability.;;; One slide called 'n.turtles' bound at 1 and any number of individuals you may want in the environment.;;; This slider allows you to manipulate the number of individuals in the environment.;;; The usual NetLogo buttons, 'setup', 'go' (forever) and 'go';;; Running this code will produce .csv files containing the contest matrices for various time points in the model's iteration.;;; THESE .CSV FILES MUST BE SORTED BY THE TURTLE.ID COLUMN PRIOR TO ANALYSES OR THE MATRICES WILL BE EVALUATED INCORRECTLY.;;;;;;;;;;;;;;;;;;;;;;;;;;;;;;;;;;;;;:::::::::::::::;;; PARAMETERS ;;;;;;;;;;;;;;;;;;;;;;;;;;;;;;;;;;;;;;;;;;;;;;;;;;;;;;;;;;;;;;;;;;;;;;;;; Below are the global parameters. Each animal has a required protein and carbohydrate intake target, ;;; ProteinIT, or Nutrient X, and CarbIT, or Nutrient Y. In the MS, these values collectively represent the intake target (IT);;; Protein is the position on the X-axis and Carbs is on the Y. Also not mentioned, ;;; but represented by sliders are the food value variables FOOD.A and FOOD.B, which determine, the slope of carb (Y) on protein (X):;;; i.e. for one unit protein intake, how much carb does a food contain. ;;; The variables FOOD.X-heading tell a turtle how to navigate through the nutrient space on the basis of one of the foods it is eating. ;;; This is a programming issue for NetLogo, but is converted to the variable;;; alpha-FOOD.X, which is the angular distance between the food being consumed and the x-axis; described as alpha-f in Senior et al. 2015.;;; The parameter EatMax determines how much a food a turtle can eat in one go, and is named as phi in the MS. ;;; Food-options is a list of the foods available (a menu), from which individuals select a food at random.;;; capacity gives the number of individual each food rail can support (as in the Senior et al. 2015);;; the abundance of food (a, in Senior et al. 2015) is specified as a slider.;;; population is a list of all individuals in the population.  globals [    ProteinIT  CarbIT  FOOD.A-heading  alpha-FOOD.A  FOOD.B-heading  alpha-FOOD.B  FOOD.C-heading  alpha-FOOD.C  EatMax  food-options  capacity  population];;;; Patches have no specified parameters, but the inbuilt location of a patch (x, y) is very important in this model.patches-own [];;; Appetite (A in the Senior et al. 2015) determines how much of a given FOOD a turtle would need to eat to maximise it's fitness on that food. ;;; The variables alpha-f and alpha-ideal as are described in the Senior et al. 2015 (and Lihoreau et al.). ;;; These values are in turn are used to calculate beta. NOTE that depending on the food being consumed, alpha-f will be alpha-FOOD.X;;; NDist and beta are the used to calculate appetite (A, as given in Senior et al. 2015 and Lihoreau et al.). ;;; Fitness (F in Senior et al. 2015) is negatively related to the euclidean distance (Edist) between the turtles current location and the intake target.;;; my-choice is a state variable, which keeps track of the individuals food-choice, ;;; in this model it can take four levels "A", "B", or "C" for foods A, B or C, and "X" of the individual has no food choice (e.g. has left a food).;;; pLeave is the probability of leaving a food having eaten it.;;; enemy is a random selected individual, that you attempt to displace when the food of choice is at capacity.;;; Qij if the probability of the ith individual displacing jth, as described in Senior et al. 2015.;;; K is the nutritional latitude value of an individual - see Senior et al. 2015.;;; my.interactions is a list of values wherein individuals record their interactions with each other member of the population.turtles-own [    appetite  alpha-f  alpha-ideal   beta  Ndist  fitness  Edist  my-choice  pLeave  enemy  Qij  K  my.interactions] ;;;;;;;;;;;;;;;;;;;;;;;;;;;;;;;;;;;;;;;;;;;;;;;;;;::;;; setup ;;;;;;;;;;;;;;;;;;;;;;;;;;;;;;;;;;;;;;;;;;;;;;;;;;;;;;;;;;;;;;;;;;;;;;;;to setup     ; Create a world    clear-all    resize-world 0 700 0 700  set-patch-size 1      ; Create an intake target of protein and Carb and make it a red-cross.  ; For simplicity I assume a balance between protein and carb is required at a (x, y) position of (500, 500).  ; One could alter these values to move the IT, although if it is increased significantly, I would recommend resizing the world.    set ProteinIT 500   set CarbIT 500  ask patches with [pxcor = ProteinIT and pycor > (CarbIT - 10) and pycor < (CarbIT + 10)] [set pcolor red]   ask patches with [pycor = CarbIT and pxcor > (ProteinIT - 10) and pxcor < (ProteinIT + 10)] [set pcolor red]    ; set the EatMax parameter. This value equates to 2, as stated in the MS. However, here is specified as the following equation   ; to prove that the model is equal to that in Lihoreau et al.   ; In Lihoreau et al, the phi is sqrt(2) / 500, and the distance between the IT and the start is 1.   ; Given phi = sqrt(2) / 500, an individual needs 353.5534 steps in a straight line to reach the IT.  ; In our model, the individual must cover a greater distance from the beginning to the IT   ; (the euclidean distance between the IT and the start point; sqrt(((0 - 500) ^ 2) + ((0 - 500) ^ 2))).   ; With a rescaled phi of 2, this is again 353.5534 number of of steps in a straight line.    set EatMax 50 ; this is equal to (sqrt 2) * ((sqrt (((0 - ProteinIT) ^ 2) + ((0 - CarbIT) ^ 2))) / 500), which rescales to be equivalent to original model in Lihoreau et al. - see Senior et al. (2015)      ; set the capacity for food rails, if an extra food is added adjust the denominator as such  ; NOTE: currently, the model only responds to the 'a' value at setup, however, one could adjust competition  ; and watch it's effects on individuals in real-time by moving this line of code to the 'go' procedure below.    set capacity (a / 3) * n.turtles    ; Create the menu of available food options, an ith food should be added here.    set food-options (list "A" "B" "C")    ; Visualise the rail for FOOD.A (based on the slider FOOD.A) as green lines by asking patches   ; associated with the correct protein:carb ratio 'turn green'. If the world is resized (e.g. if the IT is moved)   ; the values of 700 below will need to be adjusted.    if(FOOD.A <= 1)[    foreach n-values 700 [?]    [      ask patch ? (? * FOOD.A) [set pcolor green]     ]  ]    if(FOOD.A > 1)[    foreach n-values 700 [?]    [      ask patch (? * 1 / FOOD.A) ? [set pcolor green]     ]  ]    ; Set the details about FOOD.A i.e. it's heading in the NetLogo world and it's alpha as described in Lihoreau et al and the MS.     if(FOOD.A <= 1)[set FOOD.A-heading [towards patch 700 (700 * FOOD.A)] of patch 0 0]  if(FOOD.A > 1)[set FOOD.A-heading [towards patch (700 / FOOD.A) 700] of patch 0 0]    set alpha-FOOD.A subtract-headings 90 FOOD.A-heading    ; Same for Food B    if(FOOD.B <= 1)[    foreach n-values 700 [?]    [      ask patch ? (? * FOOD.B) [set pcolor green]     ]  ]    if(FOOD.B > 1)[    foreach n-values 700 [?]    [      ask patch (? * 1 / FOOD.B) ? [set pcolor green]     ]  ]    if(FOOD.B <= 1)[set FOOD.B-heading [towards patch 700 (700 * FOOD.B)] of patch 0 0]  if(FOOD.B > 1)[set FOOD.B-heading [towards patch (700 / FOOD.B) 700] of patch 0 0]    set alpha-FOOD.B subtract-headings 90 FOOD.B-heading    ; Same for Food C    if(FOOD.C <= 1)[    foreach n-values 700 [?]    [      ask patch ? (? * FOOD.C) [set pcolor green]     ]  ]    if(FOOD.C > 1)[    foreach n-values 700 [?]    [      ask patch (? * 1 / FOOD.C) ? [set pcolor green]     ]  ]    if(FOOD.C <= 1)[set FOOD.C-heading [towards patch 700 (700 * FOOD.C)] of patch 0 0]  if(FOOD.C > 1)[set FOOD.C-heading [towards patch (700 / FOOD.C) 700] of patch 0 0]    set alpha-FOOD.C subtract-headings 90 FOOD.C-heading    ; Create turtles set their nutritional states as (x, y) = (0, 0).   ; Size is arbitrary. No food decision has yet been made (my-choice = "X"), thus alpha-f is 0  ; alpha-ideal is the angle towards the IT. Colors are used to help differentiate and improve visualisation  ; Orange individuals are those with food, blue are those not located at a food and red are individuals that reach the actual IT.  ; here animals are set blue, as they have no food yet.  ; Edist is scaled to 1, to keep the fitness function equivalent to that in Lihoreau et al. (where the distance from initialisation to the IT is 1).  ; Ndist is the distance (in NetLogo units) from the current location to the IT.  ; K is fixed at 0.5.    crt n.turtles [    set xcor 0    set ycor 0    set size 10    set my-choice "X"    set heading 0    set alpha-f 0    set color blue    set alpha-ideal subtract-headings 90 towards patch ProteinIT CarbIT    set Edist (sqrt (((xcor - ProteinIT) ^ 2) + ((ycor - CarbIT) ^ 2))) / (sqrt (((0 - ProteinIT) ^ 2) + ((0 - CarbIT) ^ 2)))    set fitness exp (-2 * Edist)    set Ndist sqrt(((ProteinIT - xcor) ^ 2) + ((CarbIT - ycor) ^ 2))    set K 0.5  ]    ; Create a list of turtles in the population.    set population sort turtles    ; All turtles create a list of counts of their interactions with others - currently 0    ask turtles[    set my.interactions n-values length population [0]  ]    ; reset the ticks counter    reset-ticks  end;;;;;;;;;;;;;;;;;;;;;;;;;;;;;;;;;;;;;;;;;;;;;;;;;;;;;; go ;;;;;;;;;;;;;;;;;;;;;;;;;;;;;;;;;;;;;;;;;;;;;;;;;;;;;;;;;;;;;;;;;;;;;;;;;;;to go    ; The turtles that are not on a food (i.e. blue) choose a food,   ; work out how much of it you want to eat, eat and re-calculate their fitness, and may be leave a food.  ; Individuals that optimise their nutritional state (i.e. reach the IT) become red.    ask turtles with [color = blue] [choose-food]  ask turtles with [color = orange] [eat]  ask turtles with [color = orange] [calc-fitness]  ask turtles with [color = orange] [leave]   ask turtles with [xcor = ProteinIT and ycor = CarbIT] [set color red]      ; Advance the ticker counter    tick     ; At certain time points record data, and after 20 iterations stop.     if (ticks = 1)[record-data]    if (ticks = 3)[record-data]  if (ticks = 5)[record-data]  if (ticks = 10)[record-data]  if (ticks = 15)[record-data]  if (ticks = 20) [    record-data    stop  ]   end;;;;;;;;;;;;;;;;;;;;;;;;;;;;;;;;;;;;;; SUB MODELS or PROCESSES ;;;;;;;;;;;;;;;;;;;;;;;;;;;;;;;;;;;;;;;;;;;;;;;;;;;;;;;;;;;;;;;;;;;;;;;;;;;;;;;;;;;;;;;;; choose-food ;;;;;;;;;;;;;;;;;;;;;;;;;;;;;;;;;;; turtles choose food with randomly, with equal probability of for each food and then remember information about that food; if that food is below capacity (minus your-self) you may record that food as your choice and store the necessary information; in your state variables. If the food is below capacity, you must fight for it. To add an ith food replicate the 'if' statements for the ith food.; additionally alter the denominator in the capacity calculation at the setup above and add it to the food-options.to choose-food    set my-choice one-of food-options    if (my-choice = "A")    [      ifelse ((count turtles with [my-choice = "A"] - 1) < capacity)        [          set heading FOOD.A-heading          set alpha-f alpha-FOOD.A          set color orange        ]        [          fight         ]    ]    if (my-choice = "B")    [      ifelse ((count turtles with [my-choice = "B"] - 1) < capacity)        [          set heading FOOD.B-heading          set alpha-f alpha-FOOD.B          set color orange        ]        [          fight         ]    ]      if (my-choice = "C")    [      ifelse ((count turtles with [my-choice = "C"] - 1) < capacity)        [          set heading FOOD.C-heading          set alpha-f alpha-FOOD.C          set color orange        ]        [          fight         ]    ]  end;;;;;;;;;;;;;;;;;;;;;;;;;;; fight ;;;;;;;;;;;;;;;;;;;;;;;;;;;;;;;;to fight    ; Select an enemy from those that are on your choice of food    set enemy one-of turtles with [my-choice = [my-choice] of myself and color = orange and who != [who] of myself]    if (enemy = nobody)   [    set enemy 0    stop  ]    ask enemy [set enemy one-of turtles with [enemy = myself]]    ; calculate the probability that you'll win the fight    set Qij 1 / (1 + exp (-25 * (fitness - [fitness] of enemy)))    ; see if you win      ifelse (random-float 1 < Qij)  [        ; if you win take the food heading - kick your enemy off and forget your enemy        set heading [heading] of enemy     set alpha-f [alpha-f] of enemy    set color orange      ask enemy [set alpha-f 0]    ask enemy [set color blue]    ask enemy [set my-choice "X"]        ; Record as a victory in my.interactions        foreach n-values length population [?] [      if (item ? population = enemy)       [        set my.interactions replace-item ? my.interactions (item ? my.interactions + 1)      ]      ]        ; Reset relevant state variables to 0        ask enemy [set enemy 0]    set enemy 0    set Qij 0      ]  [        ; If you loose forget your food choice        set my-choice "X"    set color blue        ; If you loose get your enemy to record the interaction as a victory against you.        ask enemy[      foreach n-values length population [?] [        if (item ? population = enemy)         [          set my.interactions replace-item ? my.interactions (item ? my.interactions + 1)        ]      ]    ]        ; Reset relevant state variables to 0        ask enemy [set enemy 0]    set enemy 0    set Qij 0  ]  end;;;;;;;;;;;;;;;;;;;;;;;;; eating  ;;;;;;;;;;;;;;;;;;;;;;;;;;;;;to eat    ; Calculate intakes in NetLogo world angles (based on 0 degrees north),   ; then convert in to the angle alpha-ideal as described in Lihoreau et al and in the MS (0 degrees is X-axis).    set alpha-ideal subtract-headings 90 towards patch ProteinIT CarbIT    ; You then caluclate your appetite; the scalar projection of the IT from your current location   ; on to the food rail you're on by taking beta - the angular difference between alpha-f and alpha-ideal.    set beta abs subtract-headings alpha-f alpha-ideal  set Ndist sqrt(((ProteinIT - xcor) ^ 2) + ((CarbIT - ycor) ^ 2))    set appetite Ndist * cos beta    ; Either eat your appetite, or eat EatMax. If you have just eaten your appetite,   ; you will likely need to see if an alternative food is 'any better' i.e. can it get you closer to the IT  ; Note you cannot move backwards so if your appetite suggests you should, look for a different food.    ifelse (appetite >= EatMax)   [    fd EatMax  ]  [    ifelse(appetite < 0)      [        set alpha-f 0        set color blue        set my-choice "X"      ]      [        fd appetite        set color blue        set my-choice "X"      ]   ]  end;;;;;;;;;;;;;;;;;; To calulate your fitness  ;;;;;;;;;;;;;;;;;;;;;;to calc-fitness    ; calculate fitness (F). Note that in Lihoreau et al. the distance between initialisation and the IT is always 1.   ; So here, for the fitness function to be equivalent, we must rescale the NetLogo distances from the current nutritional state to the IT,   ; by the total distance between the IT and the point of initialisation.    set Edist (sqrt (((xcor - ProteinIT) ^ 2) + ((ycor - CarbIT) ^ 2))) / (sqrt (((0 - ProteinIT) ^ 2) + ((0 - CarbIT) ^ 2)))    set fitness exp (-2 * Edist)  end;;;;;;;;;;;;;;;;;;;;;;;;;;;;;;;; leave ;;;;;;;;;;;;;;;;;;;to leave    ; Calculate you probability for leaving the food.  ; if you leave set your choice as 'X', your color as blue and the food rail you're on (alpha-f) as 0.    ifelse(appetite >= EatMax)  [set pLeave (1 - K) * ((abs ((alpha-ideal * (pi / 180)) - (alpha-f * (pi / 180)))) / (pi / 2))]  [set pLeave (1 - K) * ((abs ((alpha-ideal * (pi / 180)) - (alpha-f * (pi / 180)))) / (pi / 2)) + K * ((EatMax - appetite) / EatMax)]    if(random-float 1 < pLeave)   [    set alpha-f 0    set color blue    set my-choice "X"   ]  end;;;;;;;;;;;;;;; record-data ;;;;;;;;;;;;;;;;;;;;;;;;to record-data    ; Delete any files that already exist with relevant name    if (file-exists? (word ticks ".Fitness.csv"))    [carefully [file-delete (word ticks ".Fitness.csv")]      [print error-message]]    ; Open the file and write the column names    file-open (word ticks ".Fitness.csv")    file-type "Turtle.ID,"  file-type "Fitness,"  foreach population [file-type word ? ","]  file-print ""  file-close    ; Ask turtles to record their information  ; NOTE: The completed output file MUST BE SORTED BY 'Turtle.ID' or the matrix will be wrong    ask turtles  [    file-open (word ticks ".Fitness.csv")    file-type (word who ",")    file-type (word fitness ",")    foreach my.interactions [file-type word ? ","]    file-print ""    file-close  ]  end;;;;;;;;;;;;;;;;;;;;;;; END OF PROGRAM ;;;;;;;;;;;;;;;;
